# Successful High-Volume Plasmapheresis in Acute Liver Failure

**DOI:** 10.7759/cureus.16143

**Published:** 2021-07-03

**Authors:** Harsimran S Brar, Apaar Dadlani, Alex M Ng

**Affiliations:** 1 Internal Medicine, University of Louisville, Louisville, USA; 2 Pulmonary and Critical Care, University of Louisville, Louisville, USA

**Keywords:** acute liver failure, high volume plasmapheresis, hvp, alf, plasmapheresis

## Abstract

Acute liver failure carries a high mortality. At present, liver transplant is the definitive treatment along with standard medical support. In the absence of or as a bridge to liver transplant, several liver assist therapies have been derived. Some of the therapies have shown short-term mortality benefits and transplant-free survival over standard medical treatment alone. High volume plasmapheresis (HVP) is one of such therapies and is readily available in hospitals. We discuss the case of a 28-year-old female who presented with acute liver failure, did not qualify for the liver transplant and successfully underwent HVP. Various regimens of plasmapheresis have been described in the literature of which we used the HVP for pre-determined three days. Our case emphasizes the importance of early initiation of HVP in an acute liver failure patient who did not qualify for liver transplant, and adds to the existing evidence of the utility of this particular type of plasmapheresis over other regimens.

## Introduction

Acute liver failure (ALF) or acute on chronic liver failure carries a very poor prognosis with 30-day mortality up to 26.7% without transplant [1]. Viral hepatitis and drug-induced hepatitis are the two most common causes of ALF worldwide. Acetaminophen is the most common offending drug, and among the viruses, Hepatitis A and Hepatitis E virus are the leading causes in developed countries [2]. Management is mostly supportive along with evaluation for candidacy for liver transplant. High volume plasmapheresis (HVP) is one of the most readily available liver assist therapies used in patients who do not qualify for transplant. We present a case of a young female with ALF of unclear etiology who successfully underwent HVP.

## Case presentation

A 28-year-old female with a history of polysubstance (cocaine, benzodiazepines, oxycodone/acetaminophen) abuse was admitted with altered mental status with last known normal one day before admission. Past medical history was significant for multiple overdose episodes. She was not on any prescribed medications. On arrival at the hospital, she was hypotensive to 89/53 mmHg, hypoxic to 74% oxygen saturation, afebrile and encephalopathic with Glasgow Coma Score of eight. She was intubated for airway protection and started on norepinephrine for hypotension that had not improved despite adequate intravenous fluid resuscitation. Complete blood count was significant for anemia (hemoglobin 11.2 mg/dL), leukocytosis (WBC 11.2 x 10³/µL) and thrombocytopenia (platelet count 115 x 10³/µL). Complete metabolic panel demonstrated poor liver and kidney function with aspartate aminotransferase (AST) 5181 U/L, alanine aminotransferase (ALT) 6633 U/L, alkaline phosphatase 92 U/L, total bilirubin 0.9 mg/dL, INR 2.7 and Creatinine 2.16 mg/dL (baseline of 0.8 mg/dL). Ammonia level at presentation was also elevated to 94 µmol/L. Urine drug screen was positive for cocaine and benzodiazepines. Salicylate, acetaminophen and ethanol levels were not elevated. Serologies for hepatitis A, B and C were negative. Infectious workup with urinalysis, blood cultures and chest x-ray, as well as computed tomography of head were unremarkable. Abdominal ultrasound with hepatic duplex demonstrated patent hepatic vasculature. Autoimmune serologies (anti-nuclear antibodies, anti-mitochondrial antibodies, anti-smooth muscle antibodies and serum immunoglobulin panel), serum ceruloplasmin, Herpes Simplex Virus and Epstein-Barr Virus tests were ordered to evaluate other causes of acute liver failure. After being admitted to the intensive care unit, intravenous N-acetyl cysteine was started.

Over the next 12 hours, while she remained on vasopressors and mechanical ventilatory support, hospital course was further complicated by oliguric renal failure and asymptomatic hyperkalemia requiring sustained low efficiency dialysis (SLED), disseminated intravascular coagulation (DIC) requiring multiple cryoprecipitate and fresh frozen plasma (FFP) transfusions, and myocardial injury believed to be due to demand ischemia in the setting of shock. Despite standard medical treatment (SMT), liver function continued to deteriorate, with AST/ALT levels exceeding 10000 U/L and total bilirubin levels exceeding 1.8 mg/dL in less than 24 hours. HVP was initiated on day two of admission since she was not a candidate for emergent liver transplant given her active illicit drug use. After the predetermined three days of HVP, AST and ALT markedly improved to 73 U/L and 125 U/L respectively, total bilirubin decreased to 1.4 mg/dL, mentation improved to her baseline, and oxygen requirement decreased. She was extubated on day five of hospitalization, however, she remained on continuous renal replacement therapy (CRRT) for oliguric renal failure.

## Discussion

According to the American Association for the Study of Liver Diseases (AASLD), acute liver failure is defined as rapid depreciation in liver function with elevated transaminases (as a marker of hepatocellular damage), coagulopathy with International Normalized Ratio (INR) ≥ 1.5, and encephalopathy (as a marker of impaired liver function) in a patient without preexisting liver disease and with an illness duration of <26 weeks [3]. Workup for underlying etiology and targeted therapy should be initiated as soon as possible due to high mortality. However, when etiology is unclear, supportive treatment becomes very crucial. Intravenous N-acetyl cysteine, oral or rectal lactulose, antibiotic therapy as indicated, vitamin K, correcting coagulopathies with FFP or cryoprecipitates in case of bleeding, frequent monitoring of glucose and electrolytes along with early initiation of enteral feeding (60 grams of proteins per day) include some of the major SMT measures recommended by AASLD [3]. Evaluation of candidates for liver transplant should be initiated early by specialists.

Liver assist therapies were originally developed as a bridge to liver transplantation and/or to help the native liver recover. However, in centers where transplant is not possible or in patients who do not qualify, assist therapies become vital. They are either artificial, in which sorbent-based systems are used without any cellular components to provide detoxification, or bioartificial, in which hepatocyte-based systems with or without sorbent materials are used. Of these modalities, plasmapheresis and molecular absorbents recirculation system (MARS) are the main artificial liver assist therapies. Plasma exchange not only removes pro-inflammatory cytokines, damage-related molecular patterns from degrading hepatocytes, and toxic metabolites like ammonia, but also replenishes coagulation factors [4-6]. In 2016, a randomized control trial (RCT) conducted by Larsen et al. compared the mortality benefits of combined HVP plus SMT to SMT alone in patients with acute liver failure, the only trial of its kind published to date [4]. Here, providers exchanged 15% ideal body weight (representing 8-12L per day) of a patient's plasma with an equivalent volume of fresh frozen plasma at a rate of 1-2L per hour on three consecutive days. The HVP arm showed a statistically significant increase in 30-day transplant free survival as well as improvement in other biochemical markers including ALT, INR, ammonia, and bilirubin. Thereafter, a systematic review in 2020 conducted by Tan et al. compared different types of plasmapheresis regimens [7]. These regimens ranged from three consecutive days of HVP to low volume plasmapheresis done on alternate days or until a clinic response was observed. Most of the procedures used FFPs as the exchange fluid, while some used FFP plus albumin. The review, however, included only one RCT, which was conducted by Larsen et al. as mentioned above. Tan et al. ultimately supported the use of HVP due to the high quality of evidence. However, due to lack of more RCTs, they were unable to recommend an optimal duration, volume, or type of plasmapheresis. The study did not favor its use in one specific etiology causing ALF.

A recently published network meta-analysis by Kanjo et al. in 2021 compared various liver support therapies to one another [8]. Included in this analysis were artificial liver assist therapies such as MARS, HVP, BioLogic-DT, charcoal hemoperfusion, exchange transfusion, and bioartificial liver assist therapy such as extra corporeal liver assist device. Based on their analysis, both MARS and HVP had significant mortality benefit in ALF as compared to SMT alone. However they failed to demonstrate a statistically significant mortality benefit of MARS over HVP or vice-versa in pairwise meta-analysis. Therefore, the high quality of evidence and ease of availability support the use of HVP as a liver assist therapy along with SMT for patients with acute liver failure.

## Conclusions

Acute liver failure carries high mortality, and early initiation of liver assist therapies along with supportive management can prove to be lifesaving. Our case adds to the existing evidence supporting the use of HVP in ALF. If such a patient is admitted, gastroenterology and nephrology services, if available, should be consulted immediately to evaluate for transplant and arrange for possible plasmapheresis. Given the scarcity of donated livers relative to the number of patients on the waiting list, and the transplant-free survival benefit of HVP, it is one of the promising modalities to treat ALF patients. Further RCTs evaluating the timing, duration, and frequency of HVP are warranted for more conclusive guidelines on administration of HVP.

## References

[1] Thanapirom K, Treeprasertsuk S, Soonthornworasiri N (2019). The incidence, etiologies, outcomes, and predictors of mortality of acute liver failure in Thailand: a population-base study. BMC Gastroenterol.

[2] Shah NJ, Royer A, John S (2021). Acute Liver Failure. https://www.ncbi.nlm.nih.gov/books/NBK482374/.

[3] Polson J, Lee WM (2005). AASLD position paper: the management of acute liver failure. Hepatology.

[4] Larsen FS, Schmidt LE, Bernsmeier C (2016). High-volume plasma exchange in patients with acute liver failure: an open randomised controlled trial. J Hepatol.

[5] Chung RT, Stravitz RT, Fontana RJ, Schiodt FV, Mehal WZ, Reddy KR, Lee WM (2012). Pathogenesis of liver injury in acute liver failure. Gastroenterology.

[6] Clemmesen JO, Kondrup J, Nielsen LB, Larsen FS, Ott P (2001). Effects of high-volume plasmapheresis on ammonia, urea, and amino acids in patients with acute liver failure. Am J Gastroenterol.

[7] Tan EX, Wang MX, Pang J, Lee GH (2020). Plasma exchange in patients with acute and acute-on-chronic liver failure: a systematic review. World J Gastroenterol.

[8] Kanjo A, Ocskay K, Gede N (2021). Efficacy and safety of liver support devices in acute and hyperacute liver failure: a systematic review and network meta-analysis. Sci Rep.

